# Coronary artery bypass grafting in Takayasu’s disease – importance of the proximal anastomosis: a case report

**DOI:** 10.1186/s13256-015-0767-5

**Published:** 2015-12-15

**Authors:** Anne Kuijer, Matthijs F. M. van Oosterhout, Geoffrey T. L. Kloppenburg, Wim J. Morshuis

**Affiliations:** 1 Department of Surgery, Diakonessenhuis Utrecht, Bosboomstraat 1, P.O. Box 80250, 3508 TG Utrecht, The Netherlands; 2 Department of Pathology, Sint Antonius Hospital Nieuwegein, Koekoekslaan 1, mailbox 2500, 3430 EM Nieuwegein, The Netherlands; 3 Department of Cardiothoracic Surgery, Sint Antonius Hospital Nieuwegein, Koekoekslaan 1, mailbox 2500, 3430 EM Nieuwegein, The Netherlands

**Keywords:** Coronary artery bypass graft, Coronary artery disease, Proximal anastomosis, Takayasu’s arteritis, Venous grafts

## Abstract

**Introduction:**

Treatment of coronary artery involvement in Takayasu’s arteritis is challenging. Coronary artery bypass grafting may be required. The use of saphenous vein grafts is recommended because of possible inflammatory involvement of the internal thoracic arteries. However, inserting the proximal anastomosis on inflamed aortic tissue may give rise to stenosis. Only a few cases of inserting a proximal anastomosis in patients with Takayasu’s arteritis have been reported in the literature. To date, no consensus has been reached on the best way to perform this procedure in patients with Takayasu’s arteritis.

**Case presentation:**

We report a case of a 25-year-old white woman with Takayasu’s arteritis who had recurrent angina after two previous treatments had failed, due to left main stem stenosis. She was successfully treated by coronary artery bypass grafting using a Dacron patch to insert the proximal anastomosis.

**Conclusions:**

We are the first to report an uncomplicated case in which a Dacron (Vascutek®, Renfrewshire) prosthetic patch was used to insert the proximal anastomosis on an inflamed aorta in a patient with Takayasu’s arteritis. The patch prevents contact between inflamed tissue and the graft, which we believe reduces the risk of graft failure. This case might inspire other thoracic surgeons in the challenging task of performing revascularization techniques in patients with an inflamed and fragile aorta.

## Introduction

Takayasu’s arteritis (TA) is an idiopathic vasculitis which mainly affects the aorta and proximal parts of its major branches. TA mostly occurs in women in their second or third decade and has a worldwide distribution, although the disease seems to have a higher incidence in Southeast Asia, South Africa and Latin America [1]. In 10 to 30 % of cases of TA there is coronary involvement, with the left coronary ostia most frequently affected, sometimes requiring coronary artery bypass grafting (CABG) [2, 3]. Surgical treatment of coronary involvement in TA is challenging due to the necessity to manipulate fragile and inflamed aortic tissue [4]. The internal mammary arteries are often affected by the disease, which may render their use unsuitable in CABG. Therefore saphenous venous grafts (SVGs) are mostly used in CABG in patients with TA [2, 3]. Severely inflamed and calcified lesions of the ascending aorta, however, make the SVG anastomosis to the aorta rather precarious. This fragile proximal anastomosis leads to increased postoperative complications such as hemorrhage and high rates of early graft failure [2, 5].

We report the case of a patient with a documented history of TA resulting in occlusion of her left main coronary artery who received a CABG in 2004: left internal thoracic artery (LITA)-left ascending artery (LAD). In 2012 she repeatedly had severe angina due to graft failure and was successfully treated by re-CABG using a Dacron (Vascutek®, Renfrewshire) patch on her aorta for the proximal anastomosis.

## Case presentation

**Fig. 1:**
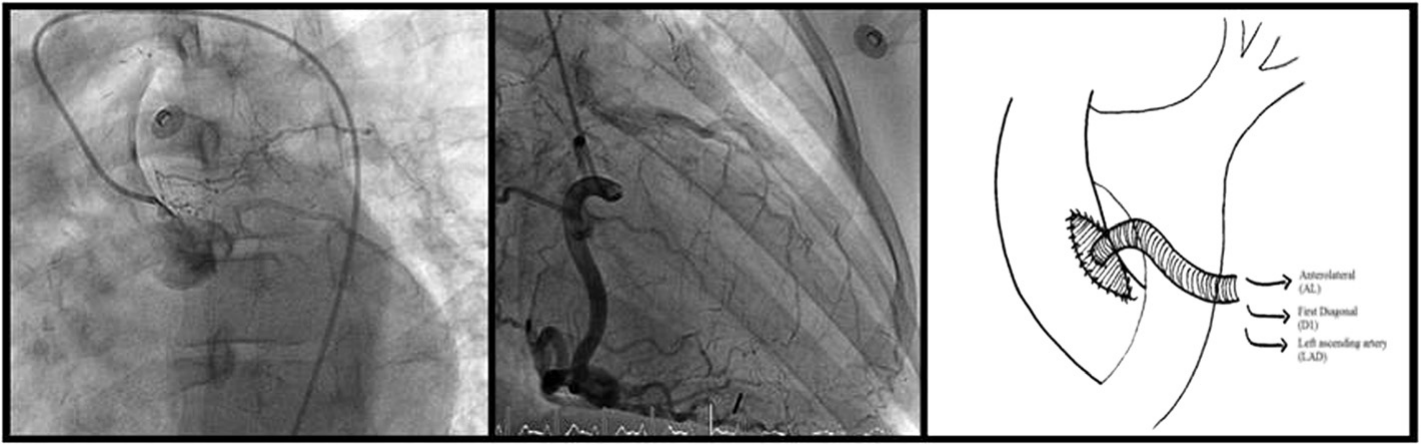
Coronary angiogram showing a total occlusion of the left system (*left*) with collateral filling from the right, dominant system (*middle*). Operation technique: a venous graft sutured into a tailor-made Dacron patch on the ascending aorta (*right*)

A 25-year-old white woman with a documented history of TA and total occlusion of her left main coronary artery received a CABG (LITA-LAD) in 2004. In 2012, two episodes of angina, due to graft failure, were treated by percutaneous coronary interventions using a Xience Prime drug-eluting stent. Unfortunately she was referred back to our hospital with signs of unstable angina after an initial symptom-free period of 4 months. During this period she received two platelet inhibitors (ticagrelor 90 mg, orally twice a day and Ascal (carbasalate calcium) 80 mg once a day), metoprolol 150 mg, ramipril 2.5 mg, simvastatin 40 mg, isosorbide mononitrate 60 mg twice a day and one dose of methotrexate (2 mg) every week in combination with folic acid. A clinical examination showed no abnormalities, besides a significant difference in blood pressure to the prejudice of the left side with absent radial pulsations. Her chest X-ray showed a normal heart size. A 12-lead electrocardiogram showed T-wave depression in the precordial and lateral leads. Blood biochemical and hematological parameters, including cardiac enzymes, were within normal range. A computed tomography angiogram was performed revealing a thickened aortic wall at the level of the aortic arch with a normal diameter and occlusion of her left subclavian and left pulmonary artery. The coronary angiogram revealed a total occlusion of her left main coronary artery and recurrent graft failure, with significant double in-stent restenosis of 99 % in the LITA-LAD. Her left coronary system was filled by collaterals from the right, dominant, system (Fig. 1).

**Fig. 2:**
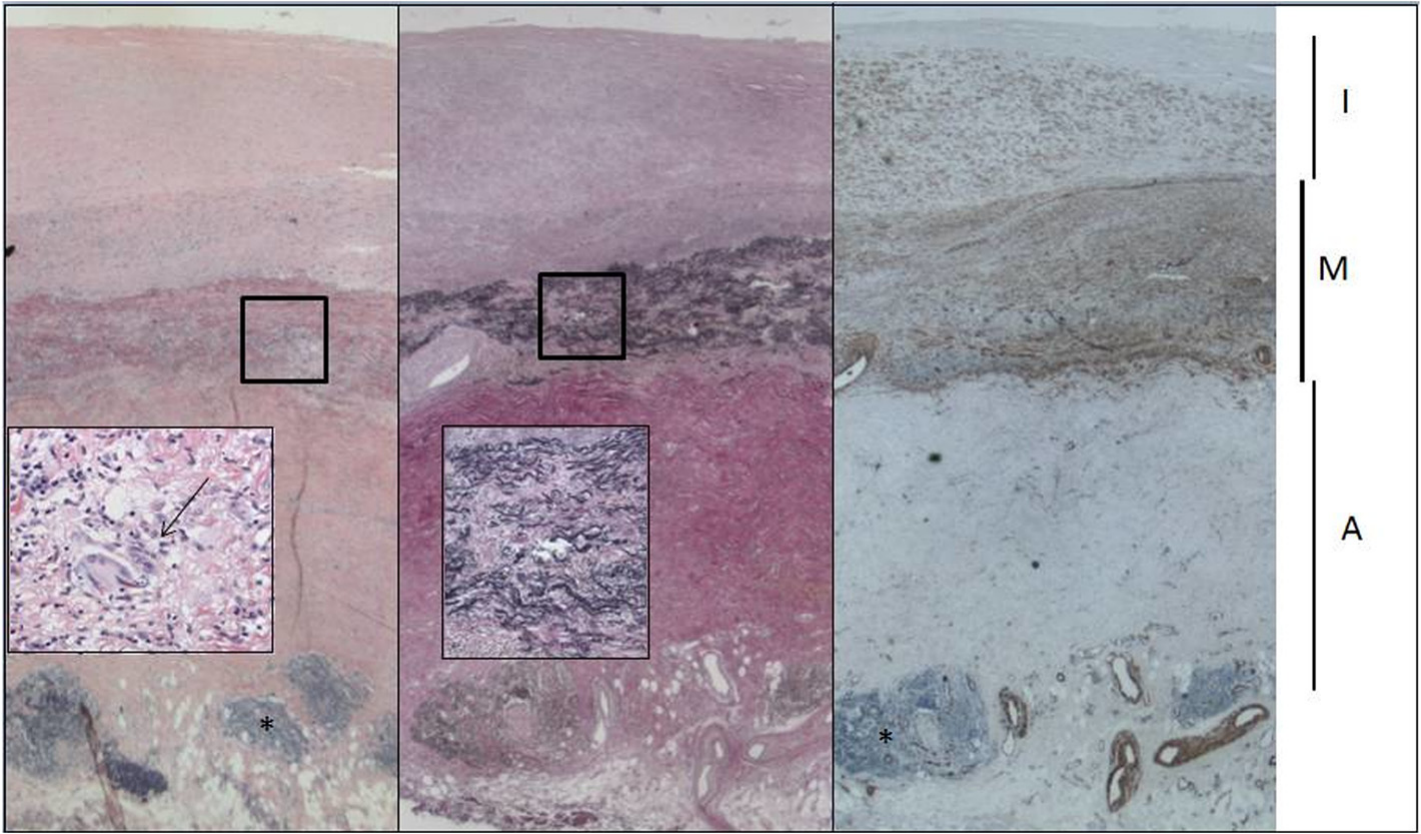
Aortic wall: stain (*left*) and Elastica van Gieson (*middle*) show the widened intima (*I*) due to proliferation of myofibroblasts, best seen in the smooth muscle actin staining (*left*). The media (*M)* is thinned with fragmentation of elastic fibers (*middle panel and inset*) associated with a lymphohistiocytic infiltrate (*left*) and a few giant cells (*inset left, arrow*). The adventitia (*A*) shows acellular collagenous fibrosis and a few nonspecific lymphoid aggregates (*)

In consultation with the patient we decided on a re-CABG using a Dacron (Vascutek®, Renfrewshire) patch on her aorta for the proximal anastomosis which was performed on 6 September 2012. Intraoperatively we encountered a thickened and inflamed aortic wall, in which the left lateral side was severely affected. We performed redo CABG using a SVG to the anterolateral, first diagonal and LAD. For the proximal anastomosis an oval Dacron (Vascutek®, Renfrewshire) patch was tailor-made by the surgeon and attached to her side-clamped aorta. Subsequently, the venous graft was sutured into the patch using prolene. Biopsies from the ascending aortic wall and the LITA proximal to the stent were taken. Histological examination of the aortic wall specimen demonstrated extensive changes including significant intima proliferation without calcification and a nonspecific chronic inflammatory response with widespread fibrosis (Fig. 2). The LITA specimen showed some reactive changes but no specific inflammatory characteristics. Both the intraoperative and postoperative courses were uneventful and the patient was discharged 5 days after surgery. Fraxiparine (nadroparin) injections (0.3 ml) were added to the medical regimen and Ascal (carbasalate calcium) dosage was increased to a 100 mg once a day.

Since then, the patient routinely visits the cardiology out-patient clinic. Her last examination (October 2015) revealed no signs of recurrent coronary perfusion problems: she reported no angina complaints during exercise, an electrocardiogram revealed no abnormalities and echocardiography showed no signs of left ventricular dysfunction or wall-motion abnormalities. Since December 2014 she stopped taking methotrexate because of an active pregnancy wish. Despite the withdrawal of immunosuppressive medication her TA is still in remission to date.

## Discussion

Coronary involvement in patients with TA is frequently seen. There are many revascularization methods such as endarterectomy, percutaneous transluminal angioplasty (PTA), drug-eluting stents, ostioplasty and CABG, of which the optimal method is yet to be determined. Since patch angioplasty and endarterectomy are associated with higher long-term restenosis rates, surgical procedures with bypass grafts seem preferable [6–8]. Coronary lesions in patients with TA are mostly located at the ostia or the proximal coronary segment of the left main coronary artery and these locations are deemed unsuitable for percutaneous techniques [9]. Unfortunately, CABG in patients with TA brings many difficulties. Due to possible involvement of the internal mammary arteries in TA, SVGs are mostly used as graft material [2, 3]. Using SVGs, restenosis rates up to 50 % have been reported, which are significantly higher compared to rates of patients with coronary disease due to atherosclerosis [10]. Occlusion of the proximal ostium of the anastomosed site, resulting from intimal hypertrophy, is the most reported late complication in patients with TA undergoing CABG [11]. To avoid graft failure and complications caused by manipulating an inflamed aorta, different techniques have been described to attach the SVG to the aorta. If possible, the proximal anastomosis should be sutured on a healthy region of the ascending aorta and the operation should preferably be performed in a non-active stage of the disease. In addition, postoperative use of steroids is recommended in patients with clinical and serological signs of active disease.

Only few cases of inserting a proximal anastomosis in patients with TA have been reported in the literature. For example Yamaguchi *et al*. reported a case were aortic connectors were employed for the proximal anastomosis in an off-pump CABG [12]. Using the connectors, the proximal anastomosis can be made faster and without clamping the ascending aorta. The procedure is impossible if there is no suitable place for the connector on a severely inflamed aorta [12]. Another way to prevent SVG occlusion caused by inflamed aortic tissue is to insert the proximal anastomosis on an autogenous pericardial patch or on a prosthetic patch [13]. Others described the use of a pericardial patch to combine coronary ostioplasty with insertion of the proximal anastomoses [14]. Despite the abovementioned techniques complication rates remain high, and there is a need for improvement. In particular, literature reports are inconclusive about the best way to insert the proximal anastomosis in CABG.

## Conclusions

No consensus has been reached on the best way to insert a proximal anastomosis on inflamed aortic tissue. We are the first to report an uncomplicated case in which a Dacron (Vascutek®, Renfrewshire) prosthetic patch was used to insert the proximal anastomosis on an inflamed aorta. The patch prevents contact between inflamed tissue and the graft, which we believe reduces the risk of graft failure.

## Consent

Written informed consent was obtained from the patient for publication of this case report and accompanying images. A copy of the written consent is available for review by the Editor-in-Chief of this journal.

## Abbreviations

CABG: coronary artery bypass grafting
LAD: left ascending artery
LITA: left internal thoracic artery
SVG: saphenous venous graft
TA: Takayasu’s arteritis
